# Vagus Nerve Stimulation for Drug Resistant Epilepsy: Clinical Outcome, Adverse Events, and Potential Prognostic Factors in a Single Center Experience

**DOI:** 10.3390/jcm11247536

**Published:** 2022-12-19

**Authors:** Ming Shan, Hongliang Mao, Hutao Xie, Yifei Gan, Delong Wu, Jian Song, Yutong Bai, Jianguo Zhang

**Affiliations:** 1 Department of Neurosurgery, The First Affiliated Hospital of Anhui Medical University, Jixi Road 218, Hefei 230022, China; 2 Department of Neurosurgery, Beijing Tiantan Hospital, Capital Medical University, No. 119 South 4th Ring West Road, Fengtai District, Beijing 100070, China; 3 First Clinical Medical College, Anhui Medical University, Meishan Road 81, Hefei 230032, China; 4 Department of Radiology, The First Affiliated Hospital of Anhui Medical University, Jixi Road 218, Hefei 230022, China; 5 Beijing Key Laboratory of Neurostimulation, Beijing 100070, China

**Keywords:** vagus nerve stimulation, drug resistant epilepsy, quality of life, seizure burden, prognostic factor

## Abstract

Objective: Vagus nerve stimulation (VNS) has been used for adjunctive treatment in drug resistant epilepsy (DRE) for decades. Nevertheless, information is lacking on possible potential prognostic factors. Our study presents the efficacy and safety of VNS with a focus on prognostic factors in 45 patients with DRE. Methods: We retrospectively evaluated the clinical outcome of 45 consecutive patients with DRE undergoing VNS implantation in The First Affiliated Hospital of Anhui Medical University between November 2016 and August 2021. Medical records were aggregated across all patient visits. Cox proportional hazards regression was used to estimate the prognostic factors. Results: Significant decrease in seizure frequency was observed after intermittent stimulation of the vagus nerve. According to the modified McHugh classification, 11 patients (24.4%) were Class I, 11 patients (24.4%) were Class II, four patients (8.9%) were Class III, 10 patients (22.2%) were Class IV, and nine patients (20.0%) were Class V. Notably, 22 patients (48.9%) were responders and four patients (8.9%) were seizure-free at the final follow-up. No significant prognostic factors were found in this cohort. Furthermore, 37 patients reported improved quality of life. Of the patients, 22 (48.9%) experienced adverse events after surgery; hoarseness, discomfort at the surgical site, and coughing were the most common. Conclusion: The results confirmed the efficacy and safety of VNS. No prognostic factors were identified.

## 1. Introduction

Epilepsy is one of the most common chronic neurological disorders, affecting more than 70 million people of all ages, races, and social status, with neurobiological, cognitive, psychological, and social consequences [1,2,3]. Drug resistant epilepsy (DRE) is defined as the failure of trials of at least two appropriately chosen, administered, and tolerated anti-seizure medications (ASMs) [4]. Surgery is an effective treatment for DRE, and includes resection, disconnection, and neuromodulation. When resection is ineffective, palliative surgery and neuromodulation therapy are advised. The efficacy and safety of VNS have been reported by many epilepsy centers [5,6,7,8]. VNS treatment has been found more effective than best medical practice (BMP) alone, with significantly improved health-related quality of life (QOL) and reduced use of hospital services in patients with DRE [9,10].

Many studies have focused on the efficacy and safety of VNS [5,6,7,8,11,12,13]. Various epilepsy centers have reached consensus in terms of efficacy, but few studies have touched on prognostic factors in VNS treatment. Arcos et al. did not find statistical differences in the factors of “age at surgery” and “duration of epilepsy” [14]. However, duration of epilepsy of less than 15 years and age at surgery less than 18 years were subgroups with a statistically better outcome in Colicchio et al.’s study [15]. The assessment results of previous studies have been inconsistent about prognostic factors, due to the heterogeneity of patients.

The objective of this study was threefold: (1) to assess the clinical outcome of VNS treatment in DRE, including changes in seizure burden, QOL, and number of ASMs, (2) to discover potential prognostic factors, and (3) to summarize adverse events that occurred during follow-up.

## 2. Materials and Methods

### 2.1. Patient Data Collection

This was an observational retrospective study through medical records and interviews on the patients with DRE who underwent VNS implantation (Cybernetics, Houston, TX, USA) between November 2016 and August 2021 at the Department of Neurosurgery in The First Affiliated Hospital of Anhui Medical University. The study was approved by the Ethics Committee of The First Affiliated Hospital of Anhui Medical University (Ethics Committee Approval code: 2019H022). Informed consent was obtained from the patients (or their guardian) before collecting patient data.

Before implantation, all patients underwent a full evaluation of epilepsy surgery including magnetic resonance imaging (MRI), video-electroencephalogram (VEEG), 18F-fluorodeoxyglucose (FDG) positron emission tomography (PET) of the brain and neuropsychology. In these postoperative patients, we developed retrospective study exclusion criteria as follows: (1) incomplete seizure data; (2) follow-up duration less than 3 months; (3) patients or guardians refused the interview. Finally, a total of 45 patients were eligible for this study.

We reviewed the baseline data including demographic information, epilepsy etiology, mean monthly seizure frequency, dominant seizure type, current ASM regimen and QOL information. At specified intervals (3, 6, 12, 24, 36, 48, and 60 months), ASM regimen, VNS parameters, seizure frequency, response time, and QOL and adverse event information was recorded in the patient’s medical records, and the modified McHugh classification was used to assess seizure outcomes. QOL was assessed and quantified according to the QOLIE-31 questionnaire, with questionnaires filled by patients or their guardians. In addition, physicians were asked to rate each patient’s overall condition as better, unchanged or worse than pre-implantation. If patients had a cognitive or intellectual dysfunction or impairment, details were provided by guardians. Based on VEEG, dominant seizure types were divided into two groups, generalized and focal, and multifocal was subsumed into generalized. The etiology of epilepsy was classified according to the latest ILAE guidelines as genetic, structural, metabolic, immune, infectious, or unknown [16]. Patients were considered as responders if they had a reduction of ≥50% in seizure frequency compared with baseline.

### 2.2. Device Implantation and Settings

All surgical procedures were performed at the Epilepsy Center of The First Affiliated Hospital of Anhui Medical University. Bipolar electrodes were placed around the left vagus nerve and connected to a programmable pulse generator, which was implanted subcutaneously below the collarbone.

Stimulation started 2 weeks after implantation, with an output current of 0.25 mA, frequency 30 Hz, on-time 30 s and off-time 5 min as the initial parameters, with magnet stimulation on demand (0.25 mA higher than device output current). Depending on patient’s toleration, the output current was increased in increments of 0.25–0.5 mA, eventually reaching the maximum intensity of 2.5 mA.

### 2.3. Statistical Analysis

Continuous variables were presented as mean ± standard deviation (SD), and categorical variables were presented as frequencies. The Student’s paired *t*-test was used for statistical comparison of continuous variables. Fisher’s exact test was used for the comparison of categorical variables. We identified the optimal cutoff value of pre-implantation number of ASMs using receiver operating characteristic (ROC) analysis to convert continuous variables to categorical variables, and stratified patients based on the following potential prognostic factors:

1. Age of onset of epilepsy: 1–51 years old;
2. Age at implant: 5–54 years old;
3. Duration of epilepsy prior to VNS: 0.25–39 years;
4. Gender: female (*n* = 15), male (*n* = 30);
5. Pre-implantation number of ASMs: ≥3 (*n* = 24), =2 (*n* = 21);
6. Dominant seizure type: generalized (*n* = 28) and focal (*n* = 17).

Different non-responder rates (lower non-responder rate means higher responder rate) were shown based on a log rank test and Cox proportional hazards regression for hazard ratios, and were visualized via Kaplan–Meier curves.

Differences were considered significant at a bilateral *p* < 0.05. Statistical analyses were conducted using the freely accessible statistical program R (version 4.1.2).

## 3. Results

### 3.1. Demographic Data

In our cohort there were a total of 45 patients (30 male, 15 female) with a mean age of 27.9 ± 11.9 years (range 5–54 years). The mean age at seizure onset was 15.5 ± 11.0 years (range 1–51 years), mean duration of epilepsy was 11.8 ± 9.6 years (range 3 months–39 years), and the range of the follow-up period was 5–62 months. In addition, the mean number of different ASMs used prior to VNS implantation was 2.7 ± 0.7. VNS implantation was performed in 6 patients (13.3%) aged less than 12 years. Based on clinical VEEG analysis, 17 patients (37.8%) had focal type of seizure dominant, and 28 patients (62.2%) had generalized seizures dominant. The most common etiology of epilepsy was structural (44.4%, *n* = 20), followed by unknown (31.1%, *n* = 14). Two cases were diagnosed with brain tumor and two with cortical atrophy. Baseline demographic data of the study are presented in Table 1. Detailed epilepsy etiologies are presented in Table 2.

### 3.2. Device Parameters

The mean parameters during the follow-up period were 1.75 mA output current, 30 Hz frequency, ON 30 s, and OFF 5 min. Mean magnet settings were 2.0 mA current output and on-time 60 s.

### 3.3. Clinical Outcomes

#### 3.3.1. Seizure Outcomes

**Figure 1:**
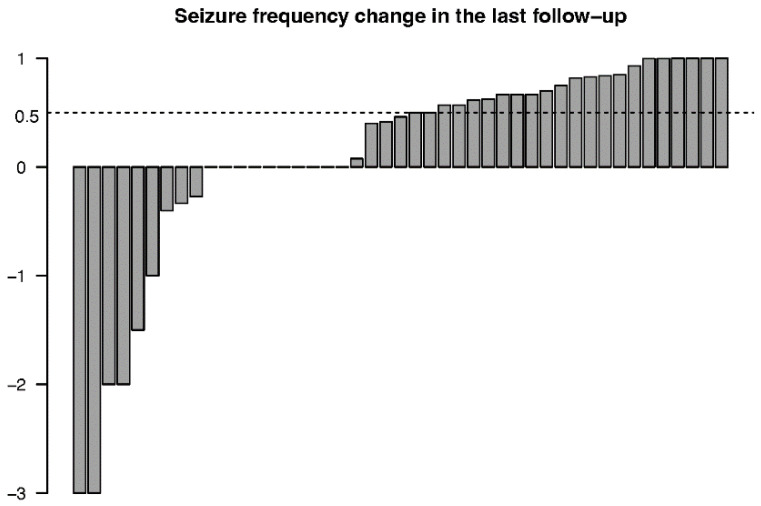
Bar chart of seizure frequency changes for every individual at the final follow-up.

The mean decreases in monthly seizure frequency after 3, 6, 12, 24, 36, 48, and 60 months were 24.2% (*n* = 45, *p* = 0.020), 36.0% (*n* = 44, *p* = 0.007), 42.3% (*n* = 34, *p* = 0.051), 45.7% (*n* = 28, *p* = 0.092), 46.3% (*n* = 20, *p* = 0.128), 58.0% (*n* = 10, *p* = 0.123), and 70.3% (*n* = 3, *p* = 0.167), respectively. The monthly seizure frequency changes for every individual at the final follow-up are presented in Figure 1. Twenty-six patients had a decrease in monthly seizure frequency, 9 patients had an increase and 10 patients had no change in monthly seizure frequency at the final follow-up. One patient who had ongoing VNS treatment for more than 3 years reported he did not perceive a significant effect from VNS and decided to stop the stimulation. Two patients had a history of epilepsy-related surgery before implantation, and neither of them obtained a satisfactory remission from seizures or decreased seizure frequency.

In terms of modified McHugh classification, 11 (24.4%) patients were identified as Class I, 11 (24.4%) as Class II, four (8.9%) as Class III, 10 (22.2%) as Class IV, and nine (20.0%) as Class V at the final follow-up; this is presented in Table 3.

Responder rates were 15.6% (*n* = 7), 26.7% (*n* = 12), 33.3% (*n* = 15), 44.4% (*n* = 20), 46.7% (*n* = 21), 48.9% (*n* = 22), and 48.9% (*n* = 22) within 3, 6, 12, 24, 36, 48, and 60 months after implantation, respectively, and the mean response time of responders was 9.9 ± 12.0 months. Four patients (8.9%) were seizure-free at the final follow-up.

#### 3.3.2. Prognostic Factors

**Figure 2:**
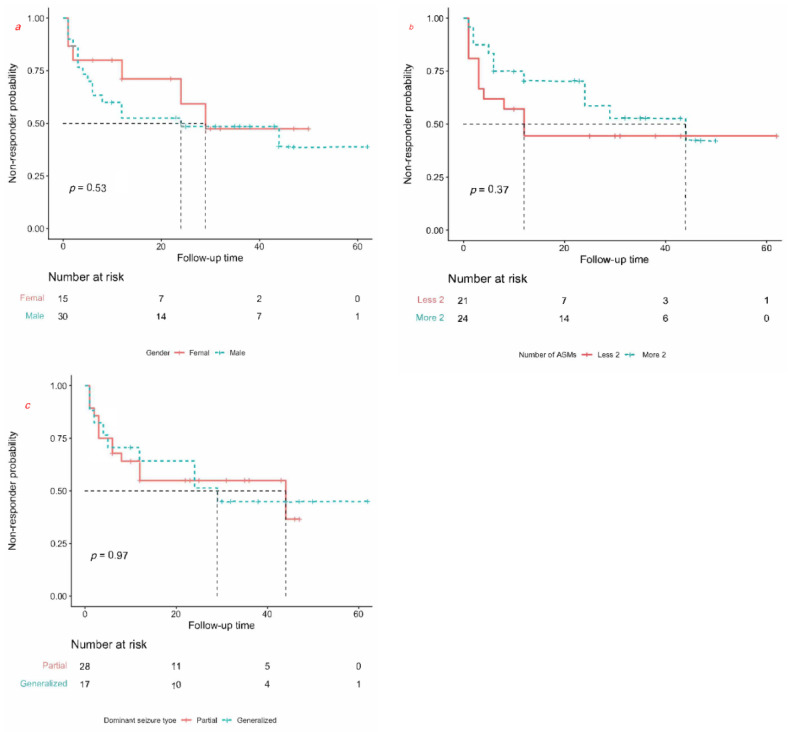
Kaplan–Meier curves for prognostic factors. Survival curve of gender (**a**). Survival curve of number of ASMs (**b**). Survival curve of dominant seizure type (**c**).

Of the three categorical variables, we did not find a significant difference for gender (*p* = 0.53, log rank test, Figure 2a), pre-implantation number of ASMs (*p* = 0.37, log rank test, Figure 2b), or dominant seizure type (*p* = 0.97, log rank test, Figure 2c). When comparing the different etiologies using Fisher’s exact test, we found no significant differences in our cohort (Table 4).

**Figure 3:**
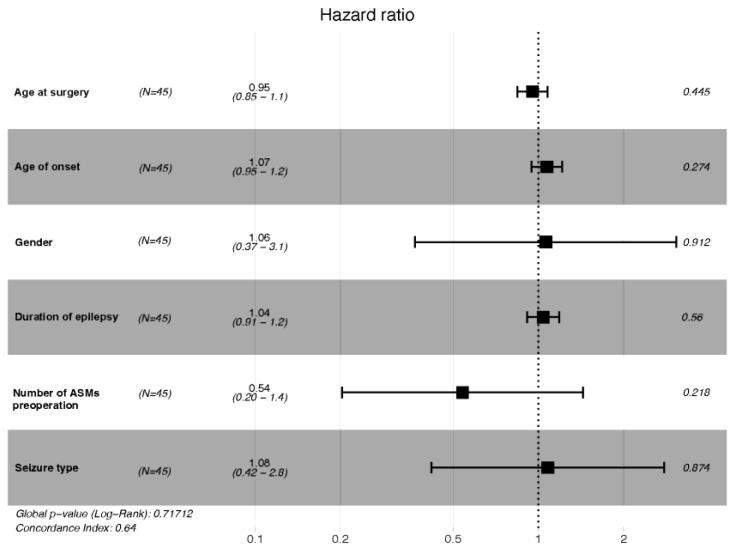
Multivariate regression analysis showing the independent prognostic factors of DRE.

Six preselected covariates were selected according to a multivariate Cox regression model with the use of a stepwise procedure. The independent prognostic factors for DRE are shown in Figure 3. However, we found no significant independent prognostic factor after the stepwise procedure was performed (global *p*-value: 0.72). The adjust hazard ratios of every variate and C-index are presented in Table 5.

#### 3.3.3. Improvement in Quality of Life

The QOLIE-31 was used to quantitatively evaluate the QOL of patients. Quite a number of patients had varying degrees of behavior disorders (44.4%, *n* = 20) and verbal communication disorders (42.2%, *n* = 19). After 3, 6, 12, 24, 36, 48, and 60 months of VNS treatment, the improvements in QOLIE-31 scores from baseline were 33.1%, 36.9%, 42.5%, 49.1%, 46.7%, 52.4%, and 54.0%, respectively.

At the final follow-up, 37, 11, and 6 patients were tagged “better”, “unchanged”, and “worse” in QOL, respectively. Among them, 27 (60.0%), 21 (46.7%), 20 (44.4%), and 6 (13.3%) patients showed improvement in ictal severity, post-seizure recovery time, seizure duration, and cognition and mood, respectively.

#### 3.3.4. Number of Anti-Seizure Medications

**Figure 4:**
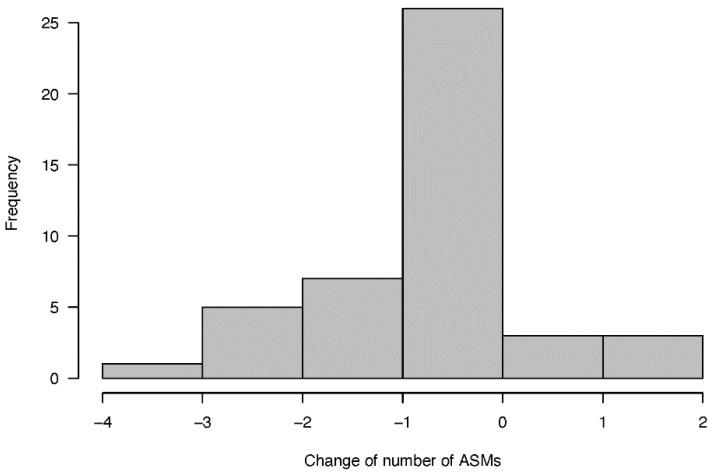
Frequency histogram of change of the number of ASMs.

The mean number of ASMs used before and after VNS implantation was 2.7 ± 0.7 and 2.4 ± 0.9, respectively, and this difference was not statistically significant (*p* = 0.125). Twenty-six patients maintained their ASM regimen, six patients had an increase in the number of ASMs they took, and 13 patients had a decrease in the number of ASMs. One patient had been off ASMs for 6 months at the final follow-up. The changes in the number of ASMs are presented in Figure 4.

#### 3.3.5. Adverse Events

Overall, the incidence of adverse events in our series was 57.8% (*n* = 26), and there were no severe complications. Discomfort at the VNS surgical site of VNS implantation was reported by 11.1% (*n* = 5), and 21 patients complained of transient hoarseness, coughing, dyspnea, nausea, cervical muscle spasm, or paresthesia when the generator was switched on. The outcomes are presented in Table 6. Most of the complications were transient and reversible, and occurred most frequently at the beginning of the stimulation treatment and less frequently during the later phase of treatment. One patient had their VNS device removed due to lack of therapeutic effect without other adverse events.

## 4. Discussion

In this study we reported on the efficacy and safety of VNS for 45 consecutive patients with DRE, 5–62 months after implantation. Nearly half (48.9%) of the patients had a decrease in overall seizure frequency, rising to over 50% at the final follow-up, and four of them (8.9%) became seizure-free. The results indicated a cumulative effect of VNS, with the responder rate increasing (from 15.6% to 48.9%) as the period of stimulation extended. Monthly seizure frequency decreased 24.2% after 3 months of stimulation and 70.3% after 60 months of stimulation compared with baseline. Our recent meta-analysis, which featured 5223 pediatric and adult patients, also confirmed the efficacy of VNS. Overall responder rates at 3, 6, 12, 24, 36, 48, and 60 months postoperatively were 42.1%, 45.5%, 40.1%, 45.1%, 48.2%, 50.2%, and 50.8%, respectively, demonstrating a tendency of increasing effectiveness with the extension of duration of stimulation [17]. Hence, we assumed that the duration of follow-up was associated with seizure reduction.

To date, there has been no conclusive evidence on prognostic factors for VNS, so we established a Cox proportional hazard ratio regression model to provide convincing results. We performed analysis of Kaplan–Meier curves to ascertain the non-responder rates among groups. We found no significant categorical prognostic factors associated with better clinical outcome. Then we conducted a Cox regression analysis (including six preselected variates) using a stepwise procedure and found that neither the duration of epilepsy, the age at onset, nor the age at surgery had an effect on clinical outcome. It is noted that there was no positive association between shorter duration of epilepsy and better response to VNS for our cohort (*p* = 0.519, LR-test). Consistent with our study findings, a shorter duration of epilepsy was not associated with a better response to VNS treatment in a cohort of 70 patients with DRE [18]. However, a meta-analysis conducted by Wang et al. found that shorter duration of epilepsy was associated with a favorable outcome for patients with DRE [19].

Age at onset (*p* = 0.180, LR-test) and age at surgery (*p* = 0.380, LR-test), also as continuous variables, were not found to be associated with prognosis. Consistent with our study, Wang et al. supposed that age at seizure onset had no significant statistical association with a favorable outcome [19]. However, in a meta-analysis that recruited 5554 patients from the VNS therapy Patient Outcome Registry and 78 clinical studies, the authors found that patients with age of epilepsy onset >12 years were more likely to be seizure-free than the younger patients, by multivariate analysis (OR, 1.89; 95% CI, 1.01–1.85) [20]. Xu et al. using a generalized linear mixed-effect model, found that earlier onset age (OR, 1.11; 95% CI, 1.06–1.16) and shorter duration of epilepsy (OR, 1.04; 95% CI, 1.00–1.07) presented a favorable clinical outcome [21].

Notably, there were no significant differences in dominant seizure type, gender, age at VNS implantation, and number of ASMs in our cohort. In terms of detailed etiology, we also found no significant prognostic factors. The detailed adjust hazard ratios of six variates are presented in Table 5. The detailed etiologies in our study were classified as post-traumatic in six patients, whose seizure frequency was 10.3 ± 5.5 per month at baseline and 0.79 ± 0.78 per month at the final follow-up, a decrease of 92.3%, and five of the post-traumatic patients were responders. Consistent with our study, a previous meta-analysis identified 74 clinical studies presenting significant benefits from VNS in post-traumatic epilepsy (a 79% reduction in seizures) [22]. This leads us to believe that VNS has a better effect on epilepsy related to brain trauma, despite a lack of significant statistical difference at the final follow-up. We also found no statistical differences for other etiologies. Meanwhile, no significant differences were observed between focal and generalized EEG outcomes in our cohort. Arcos et al. performed an observational retrospective study that enrolled 40 patients with DRE and found no significant difference between focal and generalized epilepsy at the last revision (*p* = 1) [14]. As with Arcos et al., Englot et al. found that seizure type was not a predictive factor [20].

In our study, the improvements in QOL were more pronounced among children than adults. In addition, eight of the 19 patients with unalleviated seizures showed subjective improvement in QOL. These results were somewhat subjective in that they were based entirely on reports from medical records that were based on interviews with parents or caregivers, rather than on standard criteria. Nonetheless, the reported improvement in QOL suggests that VNS treatment may have a meaningful impact on QOL in patients with VNS, independent of a reduction in seizures. The PuLsE study confirmed that VNS plus BMP was highly associated with a significant improvement in QOL compared with BMP alone [23].

In general, we did not find a statistically significant reduction in the number of ASMs that patients with DRE were taking. Thirteen patients had varying degrees of reduction in number of ASMs during the follow-up period. Only one patient discontinued all ASMs, with this response occurring at 29 months post-implant. This result was consistent with previous uncontrolled studies, and the reduction of seizure frequency may be attributed to long-term VNS and ASM interactions [24,25].

No severe or life-threatening adverse events were observed in our study. Nearly half of the patients experienced adverse reactions after surgery, the most common being hoarseness (22.2%), discomfort at the surgical site (11.1%), and coughing (8.9%). Most adverse events were transient and frequently included hoarseness and/or coughing during the “on” period. One patient in our series had the stimulator removed because it was ineffective.

This study had several limitations. First, it was a single center retrospective study and thus subject to the limitations of single center and retrospective approaches. Second, we recruited more males (30/15), and thus a gender bias may have been present, although as far as we know gender does not affect the efficacy of VNS [26]. Finally, the small sample size was a limitation. Hence, the results need to be further verified by future large prospective studies.

## 5. Conclusions

This study confirmed the efficacy and safety of VNS in treating DRE, and also confirmed that the use of VNS leads to significant improvements in seizure burden, QOL, ictal severity, and burden on parents of children with epilepsy. No baseline characteristics were associated with prognosis. Large prospective randomized trials are needed to potentially expand the number of patients who could benefit from such palliative therapy.

## Figures and Tables

**Table 1:** Demographic and clinical features of VNS patients (*n* = 45).

Male/Female; *n* (%)	30/15 (67.3%/32.7%)
Age at seizure onset (years); average ± SD	15.5 ± 11.0
Age at VNS implantation (years); average ± SD	27.9 ± 11.9
Duration of epilepsy (years); average ± SD	11.8 ± 9.6
Duration of follow-up period (months); range	5.0–60.0
Etiology; *n* (%)	
Genetic	0 (0)
Structural	20 (44.4%)
Infectious	11 (24.4%)
Metabolic	0(0)
Immune	0 (0)
Unknown	14(31.1%)
Dominant seizure type; *n* (%)	
Focal	17 (37.8%)
Generalized	28 (62.2%)
History of epilepsy-related surgery; *n* (%)	
Yes	2 (4.4%)
No	43 (95.6%)
Number of ASMs prior to VNS implantation; *n* (%)	
≥3	24 (53.3%)
2	21 (46.7%)

**Table 2:** Epilepsy etiology details of all VNS implanted patients.

Etiology of Epilepsy	*n*
Post-traumatic	6
Meningoencephalitis	11
Brain tumor	2
Perinatal hypoxic ischemic encephalopathy	1
Hippocampal sclerosis	3
Cortical atrophy	2
Encephalomalacia foci	6
Unknown	14

**Table 3:** Outcome of seizure control by modified McHugh classification at the final follow-up.

McHugh Classification	Description	Number (%)
I	80–100% reduction in seizure frequency	11 (24.4%)
II	50–79% reduction in seizure frequency	11 (24.4%)
III	<50% reduction in seizure frequency	4 (8.9%)
IV	Magnet benefit only	10 (22.2%)
V	No benefit	9 (20.0%)

**Table 4:** Responders according to etiology.

Etiology	Responder (*n* = 22)		Total (*n* = 45)	Fisher’s Exact Test
	Yes	No		
Post-traumatic	5	1	6	*p* = 0.096
Meningoencephalitis	6	5	11	*p* = 0.738
Brain tumor	0	2	2	*p* = 0.489
Perinatal hypoxic ischemic encephalopathy	0	1	1	*p* > 0.999
Hippocampal sclerosis	2	1	3	*p* = 0.608
Cortical atrophy	0	2	2	*p* = 0.489
Encephalomalacia	1	5	6	*p* = 0.187
Unknown	8	6	14	*p* = 0.530

**Table 5:** Adjust hazard ratio and C-index of the multivariate Cox regression.

	Crude HR (95 CI%)	Adjust HR (95 CI%)	*p* (Wald’s Test)	*p* (LR-Test)
Age at surgery	1.01 (0.98, 1.04)	0.95 (0.85, 1.08)	0.445	0.380
Age at onset	1.02 (0.99, 1.05)	1.07 (0.95, 1.22)	0.274	0.180
Gender	0.74 (0.29, 1.9)	1.06 (0.37, 3.08)	0.912	0.912
Duration of epilepsy	0.99 (0.94, 1.04)	1.04 (0.91, 1.19)	0.560	0.519
Number of ASMs	0.68 (0.29, 1.56)	0.54 (0.2, 1.44)	0.218	0.216
Seizure type	1.02 (0.43, 2.41)	1.08 (0.42, 2.78)	0.874	0.874

C-index: 0.64.

**Table 6:** Postoperative adverse events.

Adverse Event	Number (*n* = 26)	Incidence (%)
Hoarseness	10	22.2%
Discomfort in surgical site	5	11.1%
Coughing	4	8.9%
Nausea	2	4.4%
Paresthesia	2	4.4%
Dyspnea	2	4.4%
Cervical muscle spasm	1	2.2%

## Data Availability

The data presented in this study are available on request from the corresponding author. The data are not publicly available due to privacy regulations regarding patients.
